# The Formation and Function of *Birnaviridae* Virus Factories

**DOI:** 10.3390/ijms24108471

**Published:** 2023-05-09

**Authors:** Andrew J. Brodrick, Andrew J. Broadbent

**Affiliations:** Department of Animal and Avian Sciences, University of Maryland, 8127 Regents Drive, College Park, MD 20742, USA

**Keywords:** *Birnaviridae*, birnavirus, infectious bursal disease virus (IBDV), liquid–liquid phase separation (LLPS), biomolecular condensate, virus factory (VF)

## Abstract

The use of infectious bursal disease virus (IBDV) reverse genetics to engineer tagged reporter viruses has revealed that the virus factories (VFs) of the *Birnaviridae* family are biomolecular condensates that show properties consistent with liquid–liquid phase separation (LLPS). Although the VFs are not bound by membranes, it is currently thought that viral protein 3 (VP3) initially nucleates the formation of the VF on the cytoplasmic leaflet of early endosomal membranes, and likely drives LLPS. In addition to VP3, IBDV VFs contain VP1 (the viral polymerase) and the dsRNA genome, and they are the sites of de novo viral RNA synthesis. Cellular proteins are also recruited to the VFs, which are likely to provide an optimal environment for viral replication; the VFs grow due to the synthesis of the viral components, the recruitment of other proteins, and the coalescence of multiple VFs in the cytoplasm. Here, we review what is currently known about the formation, properties, composition, and processes of these structures. Many open questions remain regarding the biophysical nature of the VFs, as well as the roles they play in replication, translation, virion assembly, viral genome partitioning, and in modulating cellular processes.

## 1. Introduction

The *Birnaviridae* is a family of viruses that have a single protein capsid and a double stranded RNA (dsRNA) genome divided into two segments [1]. Birnaviruses have been isolated from birds, fish, and insects such as mosquitoes and flies, and the sequences of birnaviruses have also been found in pigs, porcupines, and the feces of bats [1,2,3,4,5,6]. Of these, infectious pancreatic necrosis virus (IPNV) of the salmonid fishes, and infectious bursal disease virus (IBDV) of phasianid birds are of the most significant economic impact to aquaculture and the poultry industry, respectively, with considerable investment being made into disease control, for example, through the use of vaccines or the engineering of more disease-resistant animals [1,2]. Despite their importance, little is known concerning the replication cycle of the *Birnaviridae*, compared to the more well-studied *Reoviridae* family, which also has a segmented dsRNA genome. Traditionally, much of our understanding of birnavirus replication was extrapolated from work with reoviruses, however, more recently, studies have suggested that birnaviruses have some features more akin to single-stranded (ss) positive-sense RNA viruses such as the *Picornaviridae*, and may represent an evolutionary bridge between dsRNA and ssRNA viruses [7]. Here, we review the literature on the properties of the *Birnaviridae* cytoplasmic replicative bodies, particularly concerning the role of liquid–liquid phase separation (LLPS) in their formation. Finally, we speculate on the likely functions of these structures during the reproductive cycle. With growing interest in using birnaviruses as vectors to protect against other diseases, and in oncolytic viral therapy, a more in depth understanding of how this understudied family of viruses interact with host cells is imperative.

## 2. Birnavirus Structure

The name “birnavirus” is a portmanteau of “bisegmented RNA virus”, and as such, the genome is composed of two segments of dsRNA (Segments A and B), which are contained within a single-layered icosahedral capsid with a T = 13 symmetry [7,8]. The larger of the two segments (Segment A) encodes a polyprotein which undergoes co-translational autocatalytic cleavage by the viral protein VP4 protease into the proteins pre-(p-)VP2 (VP2 ultimately forms the capsid), VP4, and VP3, which is a protein with multiple functions [9,10,11]. Segment A also contains an overlapping secondary open reading frame (ORF), encoding VP5, which may play a role in modulating virulence and the non-lytic egress of viruses [12,13,14]. The smaller of the two segments (Segment B) encodes VP1, the viral RNA-dependent RNA polymerase (RdRP) [15,16].

**Figure 1:**
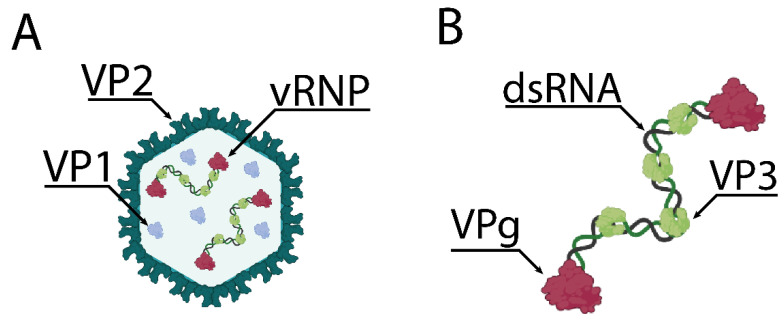
Simplified model of the IBDV virion and vRNP. (**A**) The IBDV virion is comprised of an icosahedral T = 13 capsid of VP2, containing genomic vRNPs and free VP1. (**B**) The structure of an IBDV genomic vRNP, comprising dsRNA associated with VP3, and VP1 linked to the 5′ end of each genomic strand (VPg). Created with BioRender (www.Biorender.com).

Within the virion, the birnavirus genome segments exist in the form of a viral ribonucleoprotein (vRNP) complex, associated with packaged VP1 and VP3, the latter of which binds to the genome by virtue of a sequence-independent RNA-binding domain [12,17]. VP3 also associates with the inner face of the VP2 capsid. VP1 exists within the capsid both in its free form, and linked to the 5′ ends of the genomic strands as VPg [18]. Moreover, while one full genome is comprised of one Segment A and one Segment B, *Birnaviridae* particles have been recovered from infected cells that contain between zero and four genome segments, and it is thought that birnaviruses undergo so-called “random packaging” where newly synthesized vRNP complexes nucleate encapsidation, rather than being packaged into pre-formed or partially formed capsids [17,19] (Figure 1).

The surface of the IBDV capsid is comprised of pentamers and hexamers of VP2 homotrimers, arranged so as to externally project a hypervariable region (HVR) containing four hydrophilic loops that are known to contain major epitopes of anti-IBDV antibodies [20,21]. As the name of this region implies, mutations are common, and are likely the result of immune selection pressure driving antigenic drift; the HVR sequence serves to distinguish between the IBDV strains, which are divided into eight different genogroups at the time of writing [22]. This propensity for mutation has hampered the development of a pan-neutralizing IBDV vaccine. In addition, the bipartite birnavirus genome is capable of reassortment during co-infection, further complicating epidemiology and disease control [23,24].

## 3. IBDV Reproductive Cycle

**Figure 2:**
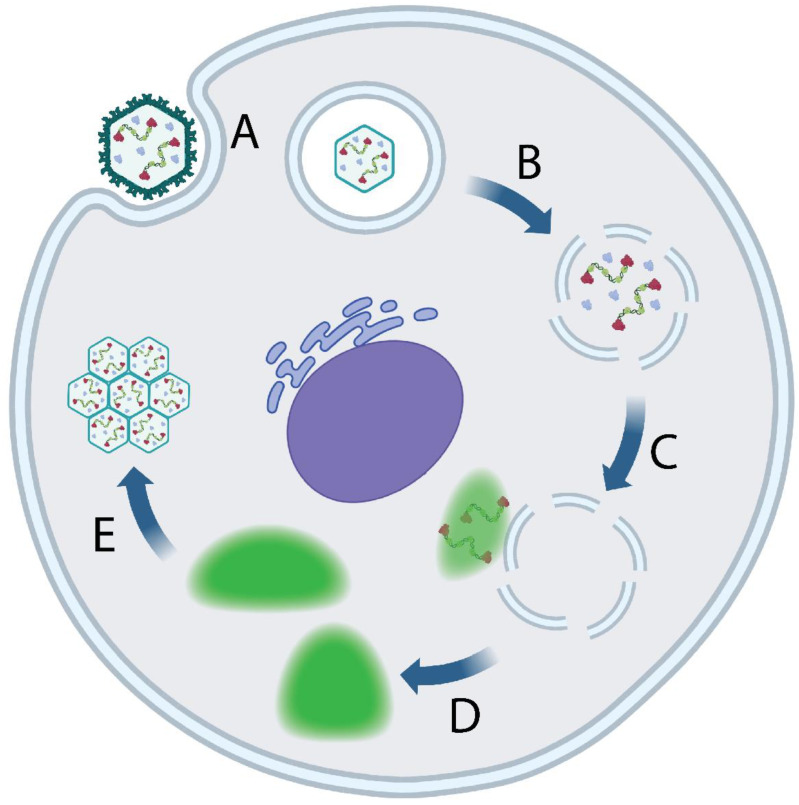
The IBDV reproductive cycle. (**A**) Viral attachment and entry, followed by uptake into an endosome. (**B**) Decreasing pH and calcium ion concentration within the endosome promotes capsid disassembly and release of pore-forming viral peptide pep46. (**C**) vRNPs exit the endosome and seed the formation of a virus factory (VF) (green), which are initially associated with the cytoplasmic leaflet of endosomal membranes. (**D**) VFs may detach from endosomal membranes and move through the cytoplasm, gradually coalescing. (**E**) Virions are assembled, and form PVAs. The mechanism of PVA formation remains unknown. Created with BioRender (www.Biorender.com).

The IBDV reproductive cycle begins with attachment of the viral particle to the host cell. The precise binding target of IBDV remains unknown, although HSP90, IgM, CD74, and CD44 have been implicated [25,26,27,28,29,30]. Following attachment, the virus is taken up into an endosome, whereupon the gradually decreasing pH and concentration of calcium ions promote the disassembly of the capsid, releasing a small peptide, pep46, which promotes pore formation in the endosomal membrane, granting the vRNP complexes access to the cytoplasm [31,32]. Cytoplasmic puncta, or inclusions, appear shortly after, notably enriched in viral proteins VP1 and VP3, as well as viral dsRNA. These structures, termed “virus factories” (VFs) or “replication complexes” are visible by fluorescence microscopy from approximately 6 h post infection (h.p.i), and are initially associated with endosomal membranes [32]. Over the course of infection, these structures grow both due to viral component synthesis and the coalescence of multiple VFs [33]. Although it has been observed that VFs are both enriched in dsRNA and are the sites of de novo RNA synthesis, unlike the replicative bodies of related reo- and rotaviruses, IBDV VFs have not been observed with virus particles inside, and the synthesis of vRNPs does not require the capsid [34,35]. Instead, transmission electron microscopy (TEM) has revealed so-called paracrystaline virus arrays (PVAs), composed of tightly packed assembled virions in the cytoplasm; it remains unknown how a VF structure leads to the formation of a PVA. It is thought that, upon the death of the host cell, virions within the cytoplasm are released into the extracellular space during cell lysis. However, IBDV-infected cells can also shed virus prior to lysis, in a process that has been reported to be mediated by protein VP5; work by Méndez et al. demonstrated the VP5-dependent appearance of membrane-wrapped PVAs, as well as a network of single-membrane vesicles containing viral particles, which may be exported from the cell [36] (Figure 2).

## 4. IBDV VF Formation

Following viral uncoating and the extrusion of the vRNP complexes into the cytoplasm, Gimenez et al. demonstrated a colocalization of VP3 with the early endosomal marker protein Rab5, a behavior further confirmed by the colocalization of VP3 with Rab5-Q79L, a constituently active Rab5 mutant that, due to overstimulated vesicular fusion, results in enlarged vesicles around which the distribution of VP3 could be readily observed. Furthermore, the authors revealed that VP3 associated with the cytoplasmic leaflet of the endosomal membrane. After treatment with saponin, which permeabilizes the plasma membrane and the internal membranes, both VP3 and the endoplasmic reticulum (ER) resident protein disulfide isomerase (PDI), which serves as a positive control for internal membrane permeabilization, were detected. However, permeabilization with digitonin, which can be made to selectively permeabilize only the plasma membrane, ablated PDI staining but did not affect VP3 detection, indicating that VP3 was exposed to the cytoplasm. This same investigation provided evidence that a VP3 region referred to as “patch 2” (P2) (residues 159, 168, 198, 200) comprised a surface-exposed, positively charged motif, which regulated the membrane associating behavior of VP3. In cells expressing VP3 with the P2 resides mutated to the negatively charged aspartic acid, colocalization of VP3 with Rab5 was lost, with the VP3 signal assuming a ubiquitous cytoplasmic distribution. This led to the hypothesis that the IBDV replication complex could be seeded on endoplasmic membranes [32].

**Figure 3:**
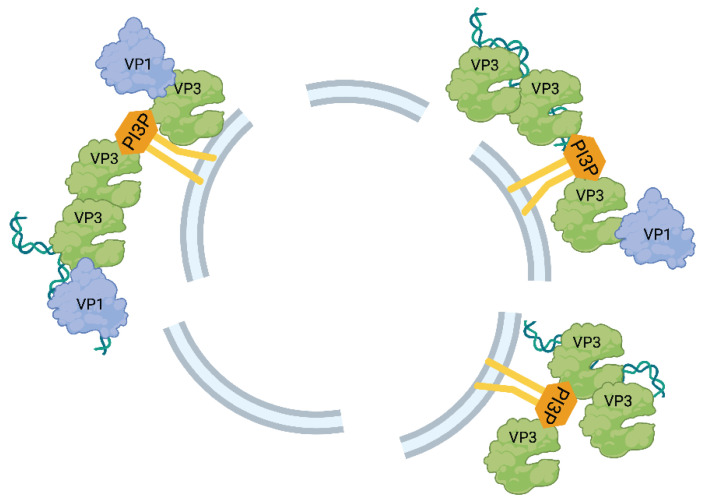
Model of IBDV VF formation in association with PI3P-enriched EEs. Following viral capsid breakdown and pep46 induced pore formation, vRNPs exit the endosome and VP3 binds PI3P molecules on the cytoplasmic leaflet of EE membranes. VP3 recruits the vRNA and other viral proteins. Further protein and RNA synthesis contribute to the growth of these EE-associated complexes. Created with BioRender (www.Biorender.com).

In the early endosome (EE) population with which VP3 was observed to colocalize, there was a particular affinity of VP3 for phosphatifylinositol-3-phosphate (PI3P)-enriched EEs, and VP3 appeared to form a non-uniform coating around PI3P-enriched EEs. This behavior was further confirmed by the treatment of VP3 expressing cells with PI3P production inhibiting the drugs LY294002 and Vps34-IN1, both of which prevented the appearance of VP3 puncta. Furthermore, treatment with rapamycin induced the detachment of VP3 puncta from the EEs by recruiting FKBP-MTM1 to the EEs, which dephosphorylated P13P. Although VP3 puncta remained visible following such treatment, there was a notable reduction in the average puncta diameter. Taken together, these data support a mechanism for IBDV VF formation based upon the nucleation at PI3P-enriched EEs, and the discontinuous VP3 “coating” of these EEs may serve as a region of enriched VP3 for the nucleation of other VF components, and ultimately the formation of the replication complex [37] (Figure 3).

## 5. IBDV VF Properties

Previous research conducted in cells infected with members of the *Reoviridae* family revealed that the viral replicative complexes are likely to be formed through LLPS, and it was subsequently hypothesized that the same might be true for the *Birnaviridae* [38,39]. In LLPS, one or more biomolecules form a network of weak homo- and heterotypic interactions that result in the formation of a distinct liquid phase within a liquid medium, analogous to the separation of other immiscible liquids [40,41]. LLPS structures are not membrane bound, yet their interior phase remains segregated from the surrounding phase, potentially via a phase-transition barrier, where repulsive interactions between “incompatible” molecules make their incursion or incorporation into LLPS structures unfavorable [42,43]. In the context of biological systems, a cellular structure comprised of biomolecules mediated by the LLPS phenomenon is sometimes termed a “biomolecular condensate”, the properties of which can be characterized by quantifying the dynamics of movement, the fluorescence recovery after photobleaching (FRAP), and the condensate break-down in response to treatment with 1,6-Hexanediol. These properties were therefore measured for IBDV VFs, to evaluate whether they were formed through LLPS [44,45,46].

First, the IBDV VFs were fluorescently tagged in vivo by the generation of recombinant IBDV viruses. Utilizing a two-plasmid reverse genetics system encoding the lab-adapted IBDV strain PBG98, a “split-GFP”-tagged virus was rescued, with the GFP-11 domain fused to the C-terminus of VP1; a tetracysteine (TC)-tagged virus was also rescued, with the TC motif fused to the same location. VFs containing VP1-GFP11 or VP1-TC could be detected in the cells either expressing GFP1-10, or in the cells stained with biarsenical dyes, respectively. Timelapse microscopy revealed that IBDV VFs were highly mobile within the cytoplasm, exhibiting movement on both small and large scales, with large-scale movement reduced upon treatment with either nocodazole or cytochalasin-D, suggesting that microtubule trafficking and the actin cytoskeleton may actively or passively (by collision) drive translocation of the structures. Furthermore, VFs were observed to undergo fission and fusion events, where a single VF splits into two or more smaller bodies, or two or more smaller VFs collide and merge into a single structure, respectively. Notably, the dynamics of the VF populations caused by multiple simultaneous infections were found to be initially independent. Cells simultaneously infected with split-GFP-labeled IBDV and TC-labeled IBDV initially developed separate populations of VFs positive for each tag. Over time, fusion of the VFs within the cell lead to the emergence of large, double-positive VFs. The role (if any) of such initially segregated VF populations and the subsequent cross-strain VF fusion in genome mixing and recombination events remains unknown [33].

Fluorescence recovery after photobleaching (FRAP) assays demonstrated that the recovery of bleached regions of split-GFP-labeled VFs in GFP1-10-expressing cells occurred on relatively short timescales (approximately 140 s for maximum recovery), suggestive of an internal liquid state. This property distinguished the IBDV VFs from protein crystals or aggregates. Moreover, the IBDV VFs also exhibited sensitivity to 1,6-Hexanediol (1,6-HD) treatment. 1,6-HD is a small aliphatic diol, which typically causes the rapid breakdown of LLPS structures, likely by perturbing the hydrogen bonding environment. As such, reactivity to this molecule is considered a standard test for LLPS, and split-GFP IBDV-infected cells treated with 4% 1,6-HD exhibited near complete loss of the VF structure within 90 s of treatment [35].

Taken together, the observations that the IBDV VFs moved rapidly in the cytoplasm, had a rapid FRAP, and were dissolved by 1,6-HD treatment are consistent with the VFs being mediated by LLPS. As the LLPS structures require the establishment of an interaction network, a sufficient concentration of critical components must be present for the structures to nucleate. If one or more of the condensate critical components have an affinity for a cellular structure or compartment, they will accumulate in the vicinity until the critical concentration is reached for the phase separation to occur, whereupon the LLPS structure could form at that site. In the context of IBDV, we hypothesize that the affinity of VP3 for PI3P-containing EE membranes causes the nucleation of the IBDV VFs at these sites, which form and grow by means of the LLPS. However, whether the VFs remain associated with these membranes throughout the reproductive cycle, or whether they dissociate and associate with other compartments is poorly understood. Recently, it has been shown that in IBDV-infected cells, VP3 structures associated with the Golgi complex, and even remained associated to the Golgi stacks after nocodazole treatment of the cells caused disruption to the native distribution of the Golgi apparatus [47]. Furthermore, these authors discovered that a Rab1b-GBF1-ARF secretory pathway axis was essential for the replication of IBDV, demonstrating that the IBDV VF structures can make contact with cellular membrane compartments to perform critical roles in virus replication. This interaction of the LLPS structures with cellular membranes remains an understudied area of cell biology [31].

## 6. IBDV VF Composition

It is known that viral dsRNA, and the proteins VP3 and VP1 strongly colocalize with VFs, however, the IBDV VP2 capsid protein was found to have a much lower colocalization, with an average Manders’ coefficient of colocalization with VP1-GFP of 0.6, compared to VP3, which was 0.9, consistent with the VFs being discrete structures to the PVAs seen by TEM [35,48]. It is known that VP3 is critical to the VF formation, as in its absence, IBDV proteins do not show a tendency to form inclusions, however, the molecular mechanisms by which VP3 forms a VF remain poorly understood [35]. It is possible that VP3 drives LLPS, however, the domains on the VP3 responsible for this phenomenon, and the mechanism by which this is achieved, remain unknown. It would be beneficial to study the nucleation of VP3 containing biomolecular condensates in a cell-free system, as has previously been conducted by Nichols et al. for the characterization of Rotavirus virus factory-like structures comprised of viral proteins NSP2 and NSP5. In this system, purified proteins were combined in DPBS buffer, leading to spontaneous nucleation. Their data comparing wild type NSP2 with the point mutant NSP2_K294E_ revealed a significant difference in both the droplet size and NSP5 concentration dependency between the two, highlighting the sensitivity of cell-free analysis methods. However, no such experiments have been performed for IBDV to date [49].

**Figure 4:**
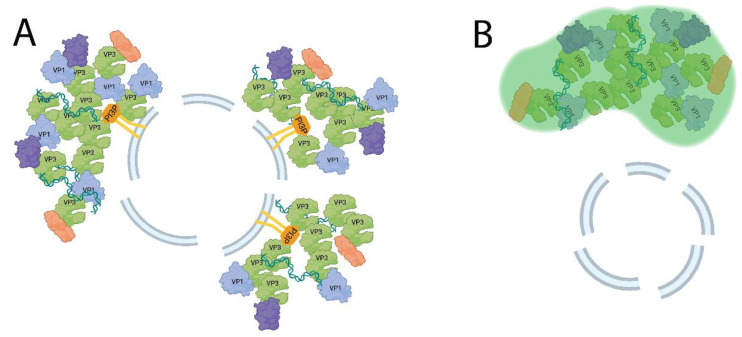
Model of IBDV VF growth. (**A**) The EE-associated VF grows due to continued viral protein and RNA synthesis. The growing VF recruits host client proteins (purple and orange). (**B**) The VF (green) may detach from the EE and move through the cytoplasm, coalescing with other VFs. Created with BioRender (www.Biorender.com).

In the terminology of LLPS, VP3 represents the condensate “scaffold”, and other viral components incorporated into the VFs are “client” biomolecules [50]. However, the range of possible client molecules recruited to a VF is not limited to those derived from the virus, and numerous condensate-forming viruses incorporate host-cell proteins into their replication complexes, for a variety of biological goals [51,52] (Figure 4). Fundamentally, viruses must utilize the host machinery to replicate, while simultaneously evading detection and antiviral responses, and the IBDV VF likely provides an environment enriched in the biomolecules and cellular proteins required for viral replication. In addition, the IBDV VF probably also shields viral components from triggering the cellular innate antiviral responses, and may also sequester the proteins involved in signaling pathways away from their intended interaction partners, preventing pathway activation in a manner similar to the strategies employed by other viruses [53,54]. It is also reasonable to hypothesize that biomolecules which normally associate with known client molecules may themselves be recruited as clients [55]. To this end, the cellular interactome of VP3 has been assayed by Co-immunoprecipitation (Co-IP) and mass spectrometry (MS). In this assay, 137 interacting proteins were identified, giving an indication as to the breadth of possible VP3 clients that may be recruited to VFs during infection. Protein–protein interaction (PPI) analysis of 121 of these proteins revealed several functionally connected groups, for example numerous mutually-interacting ribosomal subunit proteins, clusters of metabolically active proteins, regulatory proteins of the actin cytoskeleton, and cardiomyocyte adrenergic signaling molecules, indicating the breadth of possible functions of the VP3-scaffolded VF interaction network [56]. However, while defining the VP3 interactome by Co-IP and mass-spectrometry provides valuable information for follow-up studies, the necessary cell lysis steps in the protocol means any LLPS structures would be lost, and different approaches are required for defining the IBDV VF composition.

In addition to defining the VP3 interactome, several studies have characterized the interaction of VP3 with individual cellular proteins: VP3 undergoes post-translational modification, including ubiquitination; and of the E3 ubiquitin ligases, TRAF6 has been observed to directly interact with VP3 and cause a significant enhancement of VP3 K11- and K33-linked ubiquitination. This enhances the stability of VP3, markedly extending the protein’s half-life [57,58]. VP3 exerted the opposite effect on TRAF6 itself: the presence of VP3 significantly reduced the level of TRAF6 detectable by western blot, while the level of TRAF6 transcription remained unchanged, suggesting that VP3 destabilized TRAF6 by promoting its autophagic degradation. As a consequence, TRAF6-mediated NFκB activation was adversely affected, attenuating the immune responses downstream of NFκB signaling [58]. This contrasts with the effect of TRIM25, a U3 ubiquitin ligase which targets VP3 for degradation [59]. Overexpression of TRIM25 has been observed to inhibit IBDV replication, by ubiquitination at Lys854. Recombinant viruses encoding a mutant VP3 that cannot be ubiquitinated at this position (VP3 K854R) exhibited enhanced replication, indicating that TRIM25 ubiquitination modulated IBDV replication through VP3 destabilization [60]. As TRIM25 was observed to colocalize with VP3-enriched VFs, the impact of the VF concentration remains an unanswered question. It is possible that the high concentration of VP3 within VFs reduces the degradation induced by K854 ubiquitination as compared to diffuse VP3, however, experiments investigating this possibility have yet to be performed.

The dsRNA genome Is also sequestered to VFs during IBDV infection, due to the sequence-independent interaction with VP3 [61,62]. This is likely to prevent the activation of chMDA5, a chicken-expressed RIG-I like receptor (RLR) that binds dsRNA and activates the interferon signaling pathway [63,64,65]. By recruiting dsRNA to the VFs, VP3 creates an environment where dsRNA–chMDA5 interactions are energetically unfavorable, and therefore shields the immunogenic genome from surveillance [66]. The sequestration of dsRNA to the VF also enables additional RNA-binding proteins to be recruited to the VFs. As an example, Staufen1 (STAU1), a host RNA-binding protein, was found to co-immunoprecipitate with VP3, and its VF localization was confirmed by immunofluorescence microscopy [67,68]. STAU1 not only bound IBDV dsRNA, but promoted viral replication by reducing chicken interferon beta (chIFN-β) promoter activity due to competition against chMDA5 for dsRNA binding, resulting in the inhibition of the RLR signaling pathway [69].

Some modifications may be removed from proteins though interacting with the VF proteins. For example, apoptosis inhibitor 5 (API5) is recruited to the VFs as a client during infection, and the interaction between VP3 and API5 was observed to reduce API5 SUMOylation, by inducing UBC9 degradation. SUMOylated API5 enhanced MDA5-mediated IFN-β activity, so the VP3-mediated reduction in API5 SUMOylation suggested an Immune evasion role for this behavior. Consistent with this hypothesis, the viral replication was enhanced in the API5 knockdown cells, as well as the cells expressing a mutated API5 (API5 K404R) lacking its SUMOylation site [70]. In another example, one component of the IFN-β pathway, TRAF3, was found to colocalize with VP3 during IBDV infection, which reduced the formation of a TBK1–TRAF3 complex, by blocking the ubiquitination of TRAF3 at K155. This interaction was mediated by a VP3 region termed “coiled-coil region 1” (CC1), comprising the VP3 residues 12–24, a region also responsible for the dissociation of the PIK3C3–BECN1 complex, and the subsequent inhibition of autophagosome formation [71]. As the ubiquitination of TRAF3 is a critical component of the IFN-β signaling pathway, the VP3-mediated TRAF3 ubiquitination blockade during IBDV infection is analogous to the immune evasion strategies of other viruses [72,73,74].

## 7. IBDV VF Processes

**Figure 5:**
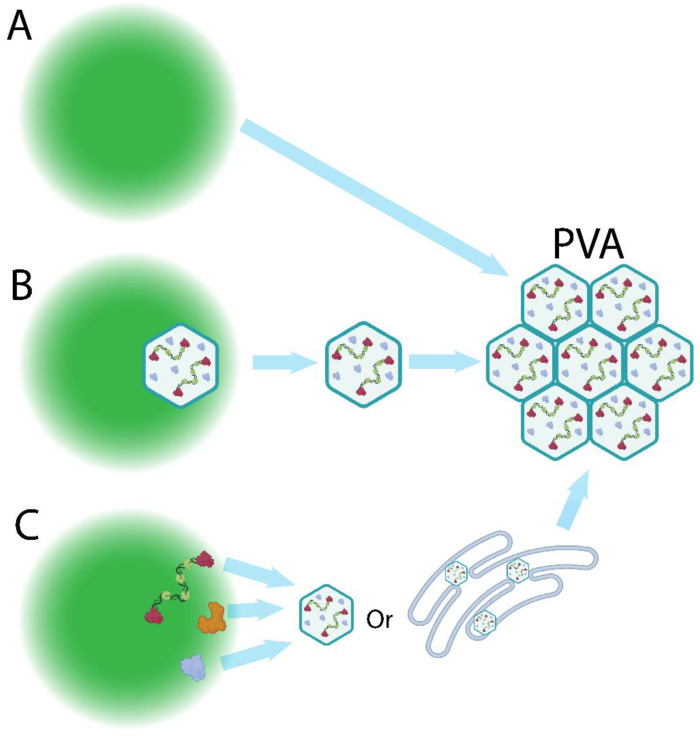
Possible mechanisms of IBDV assembly. (**A**) Simultaneous assembly–VFs (green) may reach a critical concentration of viral components, and spontaneously form virions (blue hexagons). (**B**) Assembly and ejection—virions may be assembled within the VF, and ejected due to an incompatibility with the VF liquid phase. (**C**) Off-site assembly—viral components (vRNPs and proteins) may be exported to the cytoplasm or another cellular compartment, where virion assembly occurs. Created with BioRender (www.Biorender.com).

The nomenclature surrounding virus-derived structures is somewhat confusing, as in different contexts, it may be advantageous to name and describe these bodies by their appearance, their physical properties, or their function. The terms “inclusion”, “puncta”, “body”, or variants thereof are based on their appearance in micrographs [53,75,76,77,78]. However, the appearance is often insufficient to describe the properties and behaviors of the structures, and so the physical and biochemical descriptors have also been used, such as “LLPS structures”, “biomolecular condensates”, or “viroplasms”, which may be appropriately applied once experiments have been performed validating that the bodies exhibit LLPS properties [79,80]. Finally, a well-studied structure may be named for its function, such as “virus factories”, “replication complexes”, or “replicative bodies” [39,81,82]. In the case of IBDV, all of these classes of descriptor can be meaningfully applied to some extent: IBDV VFs have a punctate appearance in infected cells, exhibit properties of liquid–liquid phase separation, and are the site of at least one step of the viral replication process, RNA synthesis. Experiments performed by Reddy et al. involved pulse-treatment with 5-ethynyl uridine (EU), a reactive RNA nucleoside analogue. RNA synthesized during the pulse period incorporates this analogue and may be detected using the Click-IT staining system [83]. Such treatment of split-GFP tagged IBDV in infected DF-1 cells revealed a strong colocalization of the de novo RNA signal with the split-GFP VF marker, suggesting the VFs to be the site of RNA synthesis [35]. While the de novo synthesized RNA detected by this protocol could be replicative intermediates or genomic strands, they could also be molecules resembling mRNA that are destined for translation. As previously discussed, ribosomal proteins have been shown to colocalize with VP3, and it is possible that translation also occurs within the VFs, although this remains to be experimentally verified [56]. The role of the VFs in viral assembly also remains poorly understood. As previously described, IBDV virions form tightly packed PVAs in infected cells. However, unlike reo- and rotaviruses, these PVAs are separate from the VFs, and the virions are not observed within VFs of IBDV, raising the question as to the location of their assembly, and the molecular mechanisms underpinning this process [35,84]. Since the viral dsRNA genome is detectable within the VFs, as are viral structural and nonstructural proteins, the necessary materials are present within VFs to assemble virions. Three potential mechanisms readily present themselves from the available data: off-site assembly, assembly and ejection, and simultaneously assembly. The off-site assembly hypothesis posits that the viral components are translocated to another cellular site, where they are assembled into completed virions. The assembly and ejection hypothesis posits that the virions are assembled within or proximal to the VFs, but are incompatible with the VF’s liquid phase, and are therefore ejected into the cytoplasm. The third mechanism, simultaneous assembly, would suggest that an entire VF may reach a critical concentration of viral components, at which time virions would rapidly and spontaneously form throughout, forming a group of virions relatively simultaneously and “converting” the VF from a viroplasm to a group of assembled viral particles. Experimental evidence favoring one of these models is, at present, lacking (Figure 5).

## 8. IBDV Interaction with Cellular Structures

The edges of IBDV VFs have been observed to colocalize with the microtubule network and the actin cytoskeleton, and nocodazole or cytochalasin D treatment inhibited VF coalescence, consistent with these structures playing a role in VF movement in the cytoplasm [33]. In addition, IBDV VFs have been found in close association with the Golgi network, even after disruption of the Golgi distribution by nocodazole treatment [85]. However, the localization of IBDV VFs with other cellular structures, for example lipid droplets, mitochondria, stress granules, etc., has not been investigated to date.

## 9. Concluding Remarks

**Figure 6:**
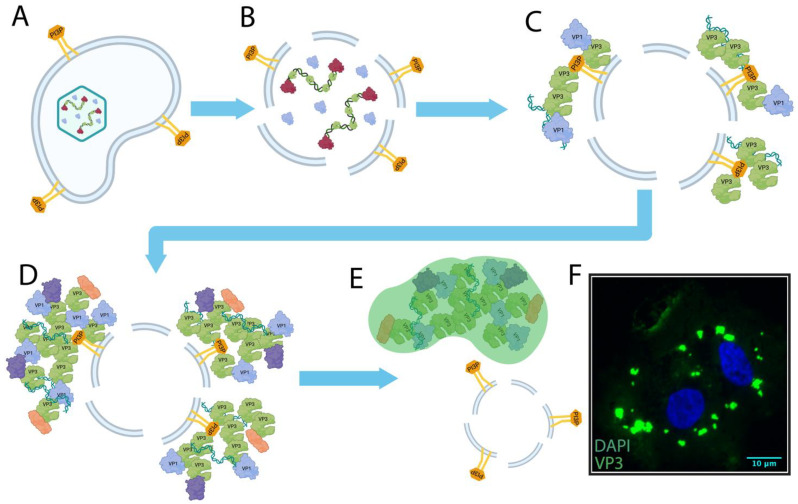
Model for nucleated IBDV VF assembly. (**A**) Following attachment and entry, intact IBDV resides within an early endosome (EE), which contains PI3P exposed to the cytoplasm. (**B**) The reduction of calcium ion concentration in the endosome induces disassembly of the IBDV capsid, exposing vRNPs comprised of VP1, VP3, and dsRNA, and freeing a viral peptide that ruptures the endosome. (**C**) IBDV VP3 molecules accumulate on the cytoplasmic leaflet of the EE by binding to PI3P. VP3 binds the VP1 polymerase and viral genome replication begins. (**D**) Continued viral genome replication, protein synthesis, and recruitment of host client proteins (purple and orange) causes the VFs to grow, and when their critical concentration is reached, they separate into a distinct liquid phase. (**E**) After nucleation, VFs might detach from EEs, and move through the cytosol. (**F**) Fluorescence micrograph of a DF-1 cell infected with IBDV strain PBG98, with cytoplasmic inclusions enriched in VP3 (VP3 green; nucleus blue). Created with BioRender (www.Biorender.com).

Despite being responsible for infections in birds, fish, and insects, and of considerable economic impact to the poultry industry and aquaculture, surprisingly little was known about the molecular mechanisms underpinning the replication of the *Birnaviridae* until the last decade. Since 2013, scientists have built a model using IBDV (Figure 6) whereby birnavirus vRNPs containing dsRNA, VP1, and VP3 are extruded into the cytoplasm upon cell entry, to nucleate the formation of VFs through the interaction of VP3 with PI3P on the cytoplasmic leaflet of membraned compartments such as EEs. In addition to binding PI3P, the VP3 protein likely forms the matrix of the VF, which is a LLPS structure that grows due to both viral component synthesis, and the coalescence together of multiple VFs. The VFs are the sites of de novo RNA synthesis, and also recruit a variety of cellular proteins, likely to provide an optimal environment for viral replication. However, many open questions remain regarding the biophysical nature of the *Birnaviridae* VFs, the molecular basis for their formation by LLPS, as well as the roles they play in replication, translation, virion assembly, and viral genome partitioning, and in modulating cellular processes. We look forward to more scientific discoveries in these areas in the future.

## Data Availability

No new data were created.
